# Gender-Related Differences in Dry Eye Symptoms Following Involutional Ectropion and Entropion Surgery

**DOI:** 10.3390/life14070815

**Published:** 2024-06-27

**Authors:** Dolika D. Vasović, Miodrag Lj. Karamarković, Milan Jovanović, Milan Stojičić, Tanja Kalezić, Milan Colić, Nikola Musić, Milan Dragišić, Miroslav Jeremić, Dejan M. Rašić, Ivan Marjanović

**Affiliations:** 1 University Eye Hospital Clinical Centre of Serbia, 11000 Belgrade, Serbia; 2 Clinic for Burns, Plastic and Reconstructive Surgery, University Clinical Centre of Serbia, 11000 Belgrade, Serbia; 3 Faculty of Medicine, University of Belgrade, 11000 Belgrade, Serbia; 4 Colic Hospital, 11000 Belgrade, Serbia; 5 Clinic of Otorhinolaryngology and Maxillofacial Surgery, University Clinical Center of Serbia, 11000 Belgrade, Serbia

**Keywords:** involutional entropion, involutional ectropion, dry eye, gender-related differences

## Abstract

This prospective case-control study investigated gender-related differences in dry eye symptoms following surgery for involutional ectropion and entropion. A total of 109 patients, aged between 65 and 89, were categorized by eyelid condition and gender. Postoperative assessments included the Tear Film Break-Up Time (TBUT) test, Schirmer I test results, corneal and conjunctival staining, eyelid margin characteristics, and scores from the Ocular Surface Disease Index (OSDI) questionnaire. The analysis revealed notable gender-related differences in dry eye manifestations. Initially, men exhibited lower TBUT scores but higher Schirmer test readings compared to women; however, these disparities diminished over time. No significant gender differences were detected in corneal and conjunctival staining, indicating similar levels of ocular surface damage across genders. Males showed significantly higher values in several eyelid margin characteristics (LMI, LMT) at various postoperative time points. According to the OSDI questionnaire, women experienced more severe symptoms of dry eye both pre- and post-operatively, suggesting a greater subjective symptom burden. When comparing surgical outcomes for ectropion and entropion, both conditions showed improvement in eyelid positioning and dry eye symptoms post-surgery. Despite these improvements, women with either condition reported more severe dry eye symptoms compared to men throughout the postoperative period. This study highlights the gender-specific variations in dry eye symptoms following eyelid malformation surgery and emphasizes the importance of adopting gender-sensitive approaches in postoperative care to improve outcomes and ocular health.

## 1. Introduction

Lower eyelid malformations, such as ectropion and entropion, significantly affect ocular health and individual well-being [1]. These conditions involve structural alterations to the lower eyelid’s position, which may disrupt the eye’s normal functioning [1]. Ectropion is a condition where the eyelid turns outward, exposing the inner eyelid surface and leading to dryness and irritation. Entropion, on the other hand, occurs when the eyelid turns inward, causing the eyelashes to rub against the cornea, which can result in discomfort and damage to the eye [1]. If left untreated, both conditions can lead to symptoms such as grittiness and dryness, intensifying discomfort and potential damage to the ocular surface. Dry eye syndrome, stemming from insufficiencies in tear production or alterations in tear composition, causes discomfort on the ocular surface and visual disturbances [2]. The health of the ocular surface relies heavily on the stability of the tear film, which provides nourishment, lubrication, and protection against environmental factors [2,3]. When tear production is inadequate or the tear composition is altered, it disrupts this delicate balance, leading to symptoms such as ocular dryness, grittiness, itching, and burning [4]. These symptoms are exacerbated under challenging environmental conditions or with prolonged use of digital devices. Moreover, dry eye can negatively impact visual acuity, causing vision to become fluctuating or blurred, and is associated with ocular surface inflammation and potential long-term damage to corneal integrity [5,6]. 

The relationship between lower eyelid malformations and dry eye is particularly complex due to their overlapping symptoms and mutual impact on the ocular surface [7,8]. Ectropion and entropion may lead to tear film instability due to compromised lid positioning, thereby increasing tear evaporation and intensifying dry eye symptoms [9,10]. Conversely, chronic dry eye conditions can induce secondary eyelid malpositions as a result of ongoing irritation and inflammation [9]. This interdependence creates a feedback loop that exacerbates ocular surface abnormalities [9,10]. Our study focuses on understanding gender differences in the outcomes of surgical correction for involutional ectropion and entropion. By tailoring interventions to individual needs, we aim to improve the quality of life and enhance overall ocular health for patients suffering from these conditions. This approach may pave the way for integrated therapeutic strategies that effectively manage both lower eyelid malformations and dry eye symptoms, ensuring optimized patient outcomes.

## 2. Materials and Methods

One hundred nine patients were included in this prospective, age-matched case-control study. Eligible patients, aged between 65 and 89 years and in good general health, were divided based on their initial diagnosis into four groups: Group A (entropion group, n = 60 eyes), Group B (ectropion group, n = 60 eyes), Group C (opposite lid entropion control group, n = 54 eyes), and Group D (opposite lid ectropion control group, n = 44 eyes). The eligibility criteria for Groups A and B included a clinical diagnosis of unilateral involutional entropion or ectropion and no history of previous eyelid surgery. Patients with a history of other significant ocular pathologies were excluded from the study. These conditions included severe ocular surface disease, glaucoma, severe blepharitis, active use of contact lenses, severe dry eye syndrome, and autoimmune conditions affecting the eyes. Additionally, patients who had undergone refractive or cataract surgery were also excluded. The study protocol was approved by the Institutional Review Board, and all patients provided informed consent before participation. The research was conducted in accordance with the principles outlined in the Declaration of Helsinki.

### 2.1. Dry Eye Tests (Tear Break-Up Time Test, Schirmer Test, Corneal and Conjunctival Staining, and Meibomian Gland Dysfunction Evaluation)

All patients underwent a comprehensive dry eye assessment before surgery and at four postoperative time points: 1, 3, 6, and 9 months after the surgical procedure. The following dry-eye tests were performed: 1. Tear Break-Up Time (TBUT): TBUT was assessed by instilling fluorescein dye into the lower fornix of the eye and measuring the interval until the first dry spot appeared on the corneal surface post-blink; 2. Schirmer I Test: Schirmer strips were placed in the lower conjunctival sac without topical anesthesia to measure the quantity of tear production over a period of 5 min, evaluating basic tear secretion; 3. Corneal and Conjunctival Staining: We utilized the Oxford Scheme for corneal and conjunctival staining to evaluate ocular surface damage in our patients. This established grading system, which ranges from 0 to 5, enabled us to systematically assess and quantify the severity of epithelial damage using fluorescein dye; 4. Meibomian Gland Dysfunction (MGD) Evaluation: Meibomian gland dysfunction was evaluated using the MGD grading system proposed by Arita et al. in 2016 [11,12]. The grading system includes the assessment of the following parameters: 1. Abnormal Lid Margin Vascularity (ALMV): Scale from 0 to 3, where 0 indicates no abnormality and 3 indicates severe vascularity or inflammation at the lid margin; 2. Plugging of Gland Orifices (PGO): Scale from 0 to 3, where 0 represents no plugging and 3 indicates significant plugging or obstruction of the gland orifices; 3. Lid Margin Irregularity (LMI): Scale from 0 to 2, with 0 indicating a smooth and regular lid margin and 2 indicating irregularities or scalloping of the lid margin; 4. Lid Margin Thickening (LMT): Scale from 0 to 2, where 0 indicates no thickening and 2 indicates pronounced thickening of the lid margin; 5. Partial Glands (PG): Scale from 0 to 3, where 0 represents no partial glands and 3 indicates a significant number of glands with partial loss or atrophy; 5. Gland Dropout (GD): Scale from 0 to 2, where 0 represents no gland dropout and 2 indicates substantial gland dropout or the loss of Meibomian glands.

### 2.2. Ocular Surface Disease Index (OSDI) Questionnaire

We employed the Ocular Surface Disease Index (OSDI) questionnaire as a valuable tool to assess subjective dry eye symptoms in patients. Higher OSDI scores indicate more severe dry eye symptoms. The OSDI questionnaire was administered to all participants before undergoing surgical correction for their respective conditions. It served as a baseline assessment of their dry eye symptoms and their impact on daily life. After the surgical procedures were completed, we conducted a final follow-up evaluation at nine months post-surgery. During this evaluation, we re-administered the OSDI questionnaire to gather data on the participants’ subjective experiences and assess any changes in their dry eye symptoms over the long term. 

### 2.3. Statistical Analysis

Statistical analysis was performed using SPSS 26.0 (IBM, Armonk, NY, USA). Since the Kolmogorov–Smirnov test did not show a Gaussian distribution of data, non-parametric tests were used. To assess gender-related differences among the four groups in dry eye symptoms, the Kruskal–Wallis test was conducted. In the event of significant differences detected by the Kruskal–Wallis test, post-hoc Dunn’s test with Bonferroni correction was employed for pairwise comparisons between groups. The Mann–Whitney U test was conducted to investigate gender-related differences within each group separately. Statistical significance was considered significant if *p* < 0.05.

## 3. Results

This study enrolled 109 patients (218 eyes) with a mean age of 74.23 ± 7.77 years. The entropion group included 57 patients (60 eyes), consisting of 31 males and 26 females. Unilateral entropion was present in 54 cases, while bilateral changes were present in 3 cases. The ectropion group included 52 patients (54 eyes), consisting of 22 males and 30 females. Unilateral ectropion was present in 50 cases, and bilateral ectropion was present in 2 cases. Groups C and D were created using the opposite eye of patients with unilateral disease. No significant difference in age or sex was found between groups (*p* > 0.05).

**Figure 1:**
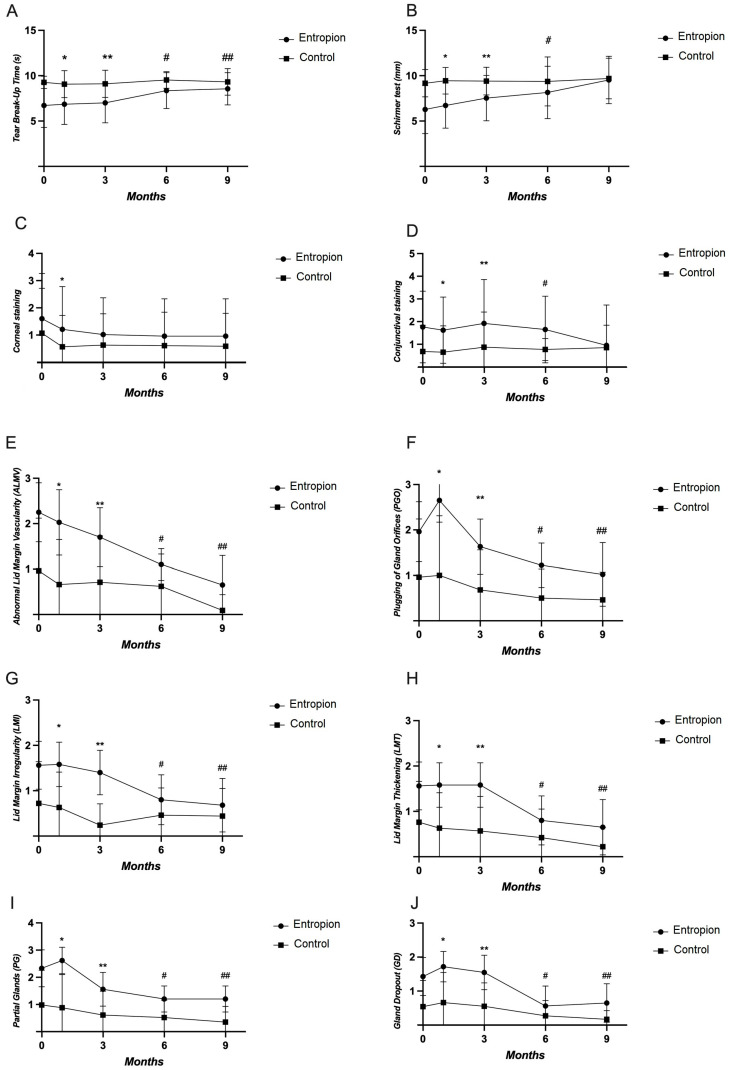
The evaluation of dry eye symptoms between the entropion side and the opposite eye at baseline, one, three, six, and nine months post-surgery. (**A**) Tear break-up time test results on the entropion side vs. the opposite eye at different time points; (**B**) Schirmer test results on the entropion side vs. the opposite eye; (**C**) Corneal staining on the entropion side vs. the opposite eye; (**D**) Conjunctival staining on the entropion side vs. the opposite eye; (**E**) Abnormal lid margin vascularity on the entropion side vs. the opposite eye; (**F**) Plugging of gland orifices on the entropion side vs. the opposite eye; (**G**) Lid margin irregularity on the entropion side vs. the opposite eye; (**H**) Lid margin thickening on the entropion side vs. the opposite eye; (**I**) Partial glands on the entropion side vs. the opposite eye; (**J**) Gland dropout on the entropion side vs. the opposite eye (* *p* < 0.05 vs. the opposite eye at one month, ** *p* < 0.05 vs. the opposite eye at three months, # *p* < 0.05 vs. the opposite eye at six months and ## *p* < 0.05 vs. the opposite eye at nine months post-op). Abbreviations: tear break-up time (TBUT); abnormal lid margin vascularity (ALMV); plugging of gland orifices (PGO); lid margin irregularity (LMI); lid margin thickening (LMT); partial glands (PG); gland dropout (GD).

The TBUT test results were significantly lower on the entropion side compared to the opposite eye at all five time points (*p* < 0.001) (Figure 1A). The Schirmer test results were significantly lower on the entropion side compared to the opposite side at baseline, one, three, and six months post-op (*p* < 0.001, *p* < 0.001, *p* < 0.001, *p* = 0.018, respectively) (Figure 1B). However, these differences were not observed at nine months post-op (*p* = 0.460). Corneal staining was significantly higher on the entropion side at baseline and one month post-op (*p* = 0.031 and *p* = 0.011, respectively), with no differences observed between sides at three, six, and nine months post-op (*p* > 0.05) (Figure 1C). Conjunctival staining was significantly higher on the entropion side compared to the opposite side at baseline, one, three, and six months post-op (*p* < 0.001, *p* < 0.001, *p* = 0.001, *p* = 0.020, respectively) (Figure 1D). The ALMV and PGO were significantly higher on the entropion side compared to the opposite side at all five time points (*p* < 0.001) (Figure 1E,F). The LMI was also significantly higher at baseline, one, three, six, and nine months post-op (*p* < 0.001, *p* < 0.001, *p* < 0.001, *p* = 0.001, *p* = 0.023, respectively) (Figure 1G). The LMT, PG, and GD were significantly higher on the entropion side compared to the opposite side at all five time points (*p* < 0.001) (Figure 1H–J).

**Figure 2:**
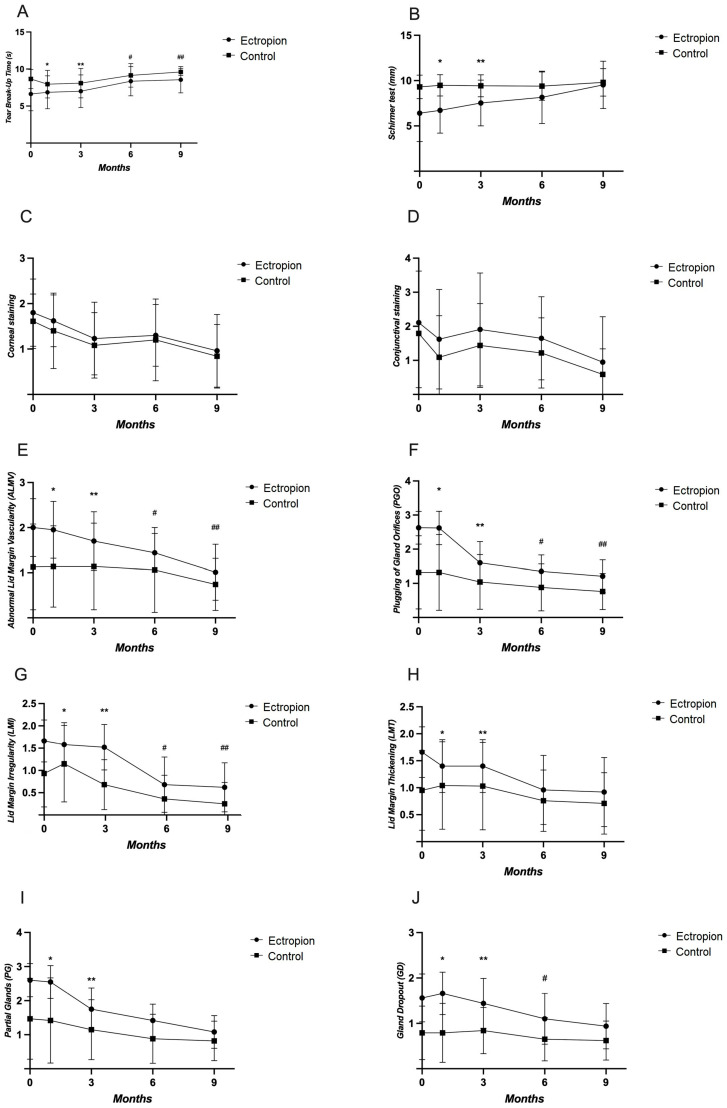
The evaluation of dry eye symptoms between the ectropion side and the opposite eye at baseline, one, three, six, and nine months post-surgery. (**A**) Tear break-up time test results on the ectropion side vs. the opposite eye at different time points; (**B**) Schirmer test results on the ectropion side vs. the opposite eye; (**C**) Corneal staining on the ectropion side vs. the opposite eye; (**D**) Conjunctival staining on the ectropion side vs. the opposite eye; (**E**) Abnormal lid margin vascularity on the ectropion side vs. the opposite eye; (**F**) Plugging of gland orifices on the ectropion side vs. the opposite eye; (**G**) Lid margin irregularity on the ectropion side vs. the opposite eye; (**H**) Lid margin thickening on the ectropion side vs. the opposite eye; (**I**) Partial glands on the ectropion side vs. the opposite eye; (**J**) Gland dropout on the ectropion side vs. the opposite eye (* *p* < 0.05 vs. the opposite eye at one month, ** *p* < 0.05 vs. the opposite eye at three months, # *p* < 0.05 vs. the opposite eye at six months and ## *p* < 0.05 vs. the opposite eye at nine months post-op). Abbreviations: tear break-up time (TBUT); abnormal lid margin vascularity (ALMV); plugging of gland orifices (PGO); lid margin irregularity (LMI); lid margin thickening (LMT); partial glands (PG); gland dropout (GD). Abbreviations: tear break-up time (TBUT); abnormal lid margin vascularity (ALMV); plugging of gland orifices (PGO); lid margin irregularity (LMI); lid margin thickening (LMT); partial glands (PG); gland dropout (GD).

The TBUT test results were significantly lower on the ectropion side than the opposite side at all five time points (*p* < 0.001, *p* = 0.007, *p* = 0.006, *p* < 0.001, *p* = 0.010, respectively) (Figure 2A). The Schirmer test results were significantly lower on the ectropion side at baseline, one, and three months post-op, with no significant difference at six and nine months post-op (*p* > 0.05) (Figure 2B). Significant differences between opposite sides were not present in corneal and conjunctival staining (*p* > 0.05) (Figure 2C,D). Conversely, the ALMV scores were significantly higher on the ectropion side compared to the opposite side at all five time points (*p* < 0.001, *p* < 0.001, *p* = 0.001, *p* = 0.039, *p* = 0.001, respectively) (Figure 2E). The PGO values were also significantly higher on the ectropion side compared to the opposite side at all five time points (*p* < 0.001, *p* = 0.006, *p* = 0.001, *p* = 0.011, *p* < 0.001, respectively) (Figure 2F). The LMI values were significantly higher on the ectropion side compared to the opposite side at all five time points (*p* < 0.001, *p* = 0.015, *p* < 0.001, *p* = 0.007, *p* < 0.001, respectively) (Figure 2G). The LMT, PG, and GD values were significantly higher on the ectropion side compared to the opposite eyelid at baseline, one month, and three months post-op (*p* < 0.05), with no significant differences at nine months post-op (*p* > 0.05) (Figure 2H–J).

**Figure 3:**
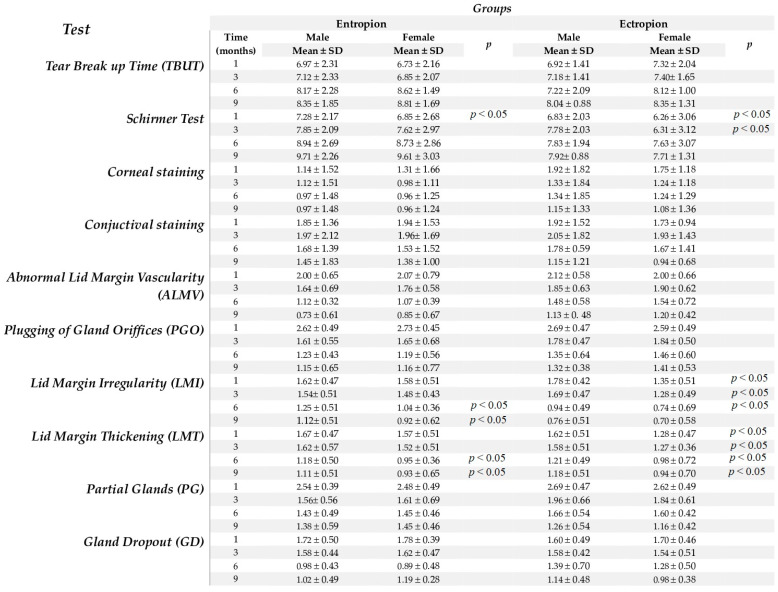
The evaluation of dry eye symptoms between males and females at baseline, one, three, six, and nine months post-surgery. Abbreviations: tear break-up time (TBUT); abnormal lid margin vascularity (ALMV); plugging of gland orifices (PGO); lid margin irregularity (LMI); lid margin thickening (LMT); partial glands (PG); gland dropout (GD).

No significant differences in the TBUT test results were found between males and females in both the entropion and ectropion groups (*p* > 0.05). The Schirmer test results were significantly higher in male vs. female patients at baseline and one month post-op in the entropion group (*p* < 0.05), and at baseline, one, and three months post-op in the ectropion group (*p* < 0.05), with no significant differences at subsequent time points (*p* > 0.05). Significant differences in corneal and conjunctival staining between males and females were not found in either group (*p* > 0.05). The ALMV, PGO, PG, and GD values showed no significant gender differences in both groups (*p* > 0.05). However, the LMI and LMT values were significantly higher in males compared to females at six and nine months post-op in the entropion group (*p* < 0.05), and LMI values were significantly higher in males at one, three, and six months post-op in the ectropion group (*p* < 0.05). The LMT values were consistently higher among males at all five time points in the ectropion group (*p* < 0.05). Furthermore, the OSDI test results were significantly higher among female participants compared to males at baseline and 12 months post-op in both groups (*p* < 0.05) (Figure 3).

## 4. Discussion

Lower eyelid malformations, such as entropion and ectropion, can profoundly impact ocular health, leading to discomfort, visual disturbances, and an increased risk of dry eye syndrome [9,10]. The surgical correction of these malformations is crucial for restoring normal eyelid anatomy, alleviating dry eye symptoms, and improving patients’ overall conditions [8,9]. Exploring gender-related differences in ocular health, especially following surgical intervention for conditions like entropion and ectropion, initiates a multifaceted discussion on how biological and possibly sociocultural factors combine to influence medical outcomes [7,8].

Gender differences in ocular surface conditions and post-surgical recovery are attributed to hormonal influences, anatomical and physiological variations, and differing symptom perceptions and reporting between males and females. Hormonal fluctuations, particularly in estrogen and androgen levels, have been shown to modulate tear production and ocular surface integrity [11]. Estrogen influences Meibomian gland function, crucial for tear film stability. The variance in hormone levels, especially in females during different life stages such as menopause, may contribute to the higher prevalence and severity of dry eye symptoms observed in female patients [11,12]. This necessitates healthcare providers to consider the endocrine profile and life stages of patients in managing post-surgical recovery. Anatomical and physiological differences between genders may also significantly influence the development of dry eye symptoms and treatment responses [10]. Structural and functional differences in eyelids, lacrimal glands, and Meibomian glands between males and females could impact the development of dry eye symptoms and response to treatment [11,12]. Studies indicate that females are more likely to report symptoms of dryness, discomfort, and visual disturbances associated with dry eye disease, reflecting a higher subjective disease burden or differences in pain perception and tolerance between genders [11,12]. Therefore, a nuanced approach to evaluating and addressing patient-reported outcomes is crucial for effective management and improving quality of life post-surgery.

The results indicate significant differences in TBUT, Schirmer test, corneal and conjunctival staining, ALMV, PGO, LMI, LMT, PG, and GD values between the affected and opposite eyes, with notable variations observed at different postoperative time points. Gender differences were observed in several parameters, with males and females showing distinct patterns in tear production and ocular surface integrity. For instance, males exhibited higher Schirmer test results early post-surgery, whereas females showed higher OSDI scores, indicating a greater subjective burden of dry eye symptoms. Moreover, males had significantly higher LMI and LMT values at various time points, suggesting more pronounced lid margin changes compared to females. The subjective burden of dry eye symptoms, as evidenced by higher OSDI questionnaire scores among females, underscores the importance of considering patient-reported outcomes alongside objective clinical assessments. Incorporating a gender-aware perspective in the clinical management of ocular surface diseases requires a comprehensive approach that accounts for hormonal influences, anatomical and physiological differences, as well as patients’ subjective experiences. Tailoring treatment and follow-up strategies to address these gender-related differences can enhance therapeutic effectiveness, reduce symptom burden, and contribute to better ocular health and patient well-being following surgery for eyelid malformations. 

The interrelationship between eyelid malformations like entropion and ectropion and their impact on ocular surface health, particularly regarding dry eye syndrome, underscores the complexity of post-surgical recovery and the importance of tailored patient care [9,10]. Several studies have investigated the impact of ocular surgery on dry eye symptoms. For example, studies on phacoemulsification have shown that surgical intervention can lead to transient dry eye symptoms due to disruption of the ocular surface and inflammatory responses [13]. Our findings align with previous research indicating a notable impact on tear film stability post-surgery. In our study, gender differences in dry eye symptoms were evident preoperatively and persisted during the postoperative period. Women exhibited higher frequencies and severities of dry eye symptoms compared to men both before and after surgery. This could be attributed to hormonal differences, which are known to influence tear film composition and stability. These gender-specific variations underscore the importance of personalized postoperative care. Monitoring these differences over time is crucial for developing effective treatment plans that address the distinct needs of male and female patients. 

In summary, the insights into gender-related differences in post-surgical outcomes for dry eye symptoms provide a compelling case for developing personalized, gender-aware strategies in ocular healthcare. Such approaches should address both the anatomical/physiological aspects of dry eye and eyelid malformations and consider patients’ subjective experiences. By integrating these considerations, healthcare providers can enhance patient satisfaction, improve clinical outcomes, and contribute to better ocular health and quality of life for patients undergoing corrective surgery for entropion and ectropion.

One of the limitations of our study is the use of traditional, invasive tests for assessing tear stability and volume, such as the Schirmer test and TBUT. These methods, while widely used, can be uncomfortable for patients and may influence the results due to their invasive nature. Non-invasive equivalent tests, such as non-invasive tear break-up time (NITBUT) and ocular surface thermography, could have provided more accurate and patient-friendly assessments. Future studies should consider incorporating non-invasive methods to enhance the precision and comfort of tear film evaluation.

## 5. Conclusions

In conclusion, this study provides valuable insights into gender-related differences in dry eye symptoms following surgery for involutional ectropion and entropion. While some gender-related differences were observed in tear film stability and tear production, other parameters related to ocular surface health and anatomical measurements showed consistent trends across genders. However, the OSDI test results indicate that female patients show lower satisfaction and more severe dry eye symptoms than males. These findings highlight the importance of considering gender as a potential factor in evaluating and managing dry eye symptoms in patients undergoing these surgical procedures.

## Data Availability

Data is contained within the article.
